# Collaborative model of care between orthopaedics and allied health professionals in knee osteoarthritis (CONNACT): process evaluation of an effectiveness-implementation hybrid randomized control trial

**DOI:** 10.1186/s12891-025-08925-0

**Published:** 2025-09-30

**Authors:** Bryan Yijia Tan, Eugene Yong Sheng Woon, Su-Yin Yang, Konstadina Griva, Soren T. Skou, David Hunter, Andrew M. Briggs, Julian Thumboo, Josip Car

**Affiliations:** 1 Woodlands Health, Singapore, Singapore; 2 Lee Kong Chian School of Medicine, Nanyang Technological University, Singapore, Singapore; 3 University of Southern Denmark, Odense, Denmark; 4 The University of Sydney, Sydney, Australia; 5 Curtin University, Perth, Australia; 6 Singapore General Hospital, Singapore, Singapore; 7 King’s College London, London, England

**Keywords:** Knee osteoarthritis, Process evaluation, Mixed methods, Hybrid trial, Implementation

## Abstract

**Objective:**

To evaluate the implementation process of a community-based, multidisciplinary intervention (CONNACT) through a randomized controlled trial (RCT) in order to contextualize the RCT outcomes and inform implementation opportunities.

**Methods:**

This study is an embedded qualitative process evaluation of the CONNACT effective-implementation hybrid RCT. Semi-structured interviews with 22 intervention patients and 14 healthcare professionals were conducted. Interviews were audio-recorded, transcribed and translated using framework analysis. Data was analysed thematically and the emergent themes were organised into the conceptual domains of RE-AIM. An explanatory sequential methods approach was used to discuss the Reach and Effectiveness domains of RE-AIM, whereby quantitative data (i.e. recruitment logs and published quantitative results) was discussed in relation to this study’s qualitative data.

**Results:**

*Reach*: 55.4% of the patients who met the inclusion criteria participated, while work or family commitments and disinterest in physiotherapy are the primary reasons for declining participation.

*Effectiveness*: CONNACT intervention is not superior to control hospital-based usual care in terms of pain, function, and quality of life, but superior in physical performance, knee satisfaction, global perceived effect and positive dietary change. The results and effectiveness of CONNACT are presented and discussed in a related publication.

*Adoption*: Healthcare professionals proposed changes for long-term sustainability (transdisciplinary approach, expert patients) despite their strong support for CONNACT.

*Implementation (context)*: A spectrum of passive-resigned and impatient-unrealistic mindsets, pain beliefs, and expectations was elucidated.

*Implementation (mechanism of impact)*: Focus on patient education and empowerment encouraged patients to actively accept their condition and practice self-efficacy. Although group classes are a source of support, motivation, and positive peer pressure, several patients preferred personalized treatments. CONNACT’s synergistic nature benefitted patients who are more complex.

*Maintenance (patient-level)*: Patients highlighted the importance of incorporating exercise into their regular routines, but lack of time and inertia remain as significant barriers.

**Conclusion:**

The themes have allowed a better understanding of the RCT primary and secondary outcomes and informed the next phase of implementation. CONNACT and similar interventions should identify and address reasons for refusing participation [Reach]; improve group classes with initial evaluations, equal attention paid to patients, tailored exercises, and acknowledge progress [Effectiveness, Implementation]; and adopt a streamlined resource-efficient transdisciplinary design [Maintenance].

**Trial registration:**

This study, which primarily employs qualitative methods for data collection, is not a clinical trial (clinical trial number: not applicable). Ethics approval (NHG DSRB ref no: 2020/00067;) was obtained before commencement of this study.

**Supplementary Information:**

The online version contains supplementary material available at 10.1186/s12891-025-08925-0.

## Background

Knee osteoarthritis (OA) is one of the largest and fastest growing causes of pain, disability and poor quality of life, particularly in the elderly [1]. International guidelines for knee OA recommend lifestyle modifications such as exercise and weight loss as first-line management [2, 3] but care for knee OA is suboptimal [4–6] and inconsistently aligned with evidence. Furthermore, there is little evidence to guide the development and implementation of effective, evidence-based chronic care models particularly in a Southeast-Asian context. To respond to this challenge, the Collaborative Model of Care between Orthopaedics and Allied Healthcare Professionals (CONNACT) for knee OA was developed in Singapore. CONNACT is a community-based, multidisciplinary, individualized 12-week program with a strong emphasis on patient activation and self-management strategies to promote long-term sustainable behavioural change. The intervention, aimed at individuals above 45 years old with symptomatic OA knee, was conducted in a community-based elder care and rehabilitation center with exercise facilities. Recently, the randomized controlled trial (RCT) evaluating the CONNACT intervention was completed, which demonstrated that CONNACT was not superior to usual hospital-based care for pain, function and quality of life, but demonstrated superior results in physical performance, knee satisfaction, global perceived effect and positive dietary change particularly over the short to medium term [7, 8]. Of note, both the CONNACT intervention and usual care (i.e. non-pharmacological management including exercise, patient education, etc.) also showed sustained improvements over a 1-year time period across a broad range of outcome measures. A prior published work, arising from the CONNACT RCT, qualitatively explored the psychologic and social factors influencing the experiences, rehabilitation, and recovery of knee OA patients [9]. The study found a pain experience is commonly influential and shared across cultures [10]; patients are willing sacrifice mobility and independence in order to preserve face; and an unwillingness to seek family assistance to maintain harmony [11] and avoid experiencing guilt [12] for burdening, worrying, or complicating their family members’ lives [13]. The CONNACT intervention is a complex intervention, which is defined by the Medical Research Council (MRC) as an intervention that contains several interacting components [14]. In line with the MRC guidance for developing and evaluating complex interventions, a process evaluation aims to complement the interpretation of the main RCT as part of a hybrid effectiveness-implementation trial [15]. The process evaluation is crucial in understanding the functioning of an intervention by examining its implementation, mechanisms of impact and context to understand how interventions work in practice and real-world settings [16]. This process evaluation utilizes the RE-AIM framework [17], which was developed to evaluate the translation of scientific advances into practice and especially public health impact and policy. In particular, it has been shown to be an appropriate model to strengthen the case for policy change and implementation for knee OA [18].

The specific aim of this embedded process evaluation was to evaluate the implementation of the CONNACT intervention using the RE-AIM framework in order to 1) understand and interpret the quantitative outcomes from the RCT through an explanatory sequential mixed methods approach, and2) inform the next phase of implementation.

## Methodology

### Design

This study is a process evaluation embedded within a larger effectiveness-implementation hybrid trial that included a RCT [8] and economic evaluation [14] to evaluate the effectiveness of the CONNACT intervention. Table 1, from the intervention’s published protocol paper [8], provides an overview of the 12-week CONNACT intervention, which the intervention patients will receive. Control patients would receive standard hospital-based care, which constituted a referral to a hospital outpatient physiotherapy service where patients were seen within two weeks upon referral [8]. The RE-AIM framework [17] and MRC domains (Table 2) were used to guide the process evaluation [16]. The quantitative primary, secondary, compliance and adverse outcomes have been reported in detail as part of the main RCT [7]. This current process evaluation predominantly reports qualtiative insights elucidated from interviews with patients and healthcare professionals. Results were reported according to the Consolidated criteria for reporting qualitative research (COREQ) guideline [19] (see Additional File 1).

**Table 1:** Intervention summary(7)

Intervention Component	Criteria to Receive Intervention	Healthcare Professional	Treatment Principles	Delivery Format
Exercise Therapy	All patients	Physiotherapist	American College of Sports Medicine (ACSM) [20] and Neuromuscular Exercise (NEMEX) [21] guidelines	Group sessions × 8
Clinical Assessment and Education (EDU)	All patients	Orthopaedic surgeon, psychologist, medical social worker	Clinical and Radiological Assessment, Pharmacological Intervention, and “Expert” Patients	Group Education sessions × 2 Support Group session × 1
Dietetics and Nutrition (NND)	BMI ≥ 23.5	Dietician	Dietary intervention/education to increase dietary-related nutrition knowledge and self-efficacy for effective weight loss [22]	Group sessions × 3
Psychological support (PSY)	PHQ-4 > 5 or PEG > 4 on all scales or PAM < 3	Psychologist, medical social worker	Acceptance and Commitment Therapy (ACT) principles [23, 24] Patient Activation [25] Pain Management Coping Strategies and improving compliance to behavioural modifications	Group sessions × 3

**Table 2:** (11) Definition and data collection methodology based on the RE-AIM criteria

Domain	CONNACT	Data collected and used (Bolded)
Reach	The absolute number, proportion, and representativeness of individuals who are willing to participate in a given initiative, intervention, or program	1. **Recruitment Log and reasons for study non-participation** 2. **Interviews with Patients and Healthcare Professionals**
Effectiveness	The impact of an intervention on important outcomes, including potential negative effects, quality of life, and economic outcomes	1. Primary (KOOS_4_) and secondary (quality of life, physical performance measures, symptom satisfaction, psychological outcomes, dietary habits, and global perceived effect) effectiveness [7]outcomes 2. Adverse outcomes 3. Economic evaluation
Adoption	The absolute number, proportion, and representativeness of settings and intervention agents (people who deliver the program) who are willing to initiate a program	1. **Interviews with Healthcare Professionals**
Implementation	At the setting level, implementation refers to the intervention agents’ fidelity to the various elements of an intervention’s protocol, including consistency of delivery as intended and the time and cost of the intervention. At the individual level, implementation refers to clients’ use of the intervention strategies	1. Compliance 2. **Interviews with Patients and Healthcare Professionals**
Maintenance	The extent to which a program or policy becomes institutionalized or part of the routine organizational practices and policies. Within the RE-AIM framework, maintenance also applies at the individual level. At the individual level, maintenance has been defined as the long-term effects of a program on outcomes after 6 or more months after the most recent intervention contact	1. 12-month effectiveness outcome measures 2. **Interviews with Patients and Healthcare Professionals**

KOOS_4_—Knee Injury and Osteoarthritis Outcome Score. KOOS4 is scored in percentages from 0 (extreme problem to 100 (no problem) [26]

#### Qualitative methods

##### Development of interview guides

A literature review was conducted to generate components for the semi-structured interview guide based on the RE-AIM framework and Medical Research Council guide to process evaluation [15]. Two separate interview guides were developed, one for patients and one for the healthcare professionals. Input on interview question design was sought from local qualitative and musculoskeletal experts experienced in the field for both semi-structured interview guides (see Additional file 2 and Additional file 3). The provision of clinical expertise and local contextualization was to ensure comprehensiveness and relevance of the interview questions to obtain insights that can achieve the study’s objectives. Both interview guides were pilot tested before implementation.

##### Purposeful sampling and recruitment

Purposeful sampling was used to identify participants for the interviews among both healthcare professionals (i.e. not involved in CONNACT) and intervention patients. Patient were purposefully selected via maximum variation sampling to include those who reported benefits and no benefits from the CONNACT intervention (i.e. minimally clinical importance difference in KOOS_4_), those who had just finished the intervention and those who were close to their final follow up at one year across a wide range of sociodemographic, languages and knee OA severity. Healthcare professionals who were either directly involved in the CONNACT intervention or from clinical disciplines relevant to CONNACT’s intervention components were sampled to obtain diversity of opinions and insights on the design, implementation, and long-term feasibility of the community-based intervention. The research coordinator, who was not involved in the delivery of the CONNACT program, contacted the eligible patients and healthcare professionals, invited them to join the study, and provided verbal explanation of the study either face-to-face or via telephone. Informed consent was obtained prior to data collection.

##### Data collection

Face-to-face interviews were conducted between April and July 2020, in a private consultation room within a tertiary care institution. The interviews were recorded and lasted between 30 and 80 min, averaging at 50 min per interview. Interviews were conducted by a male interviewer (EW) who had prior experience in qualitative methodology and the conduct of semi-structured interviews and is effectively bilingual in English and Mandarin. No prior relationship was established between the interviewer and interviewees. Field memos were made during and/or after the interviews to record situations, ambience, and non-verbal communications, as well as the interviewer’s thoughts, analytical notes, impressions, and potential biases that could contribute to data analysis. Each interview was transcribed and translated verbatim. The accuracy of the transcripts and translations were verified by comparing them against the recordings. Transcripts were not returned to the participants for comments and correction as they were contacted only once for the study and care was taken to clarify and follow up extensively on points raised by the participant during the interview.

##### Data analysis

**Fig. 1:**
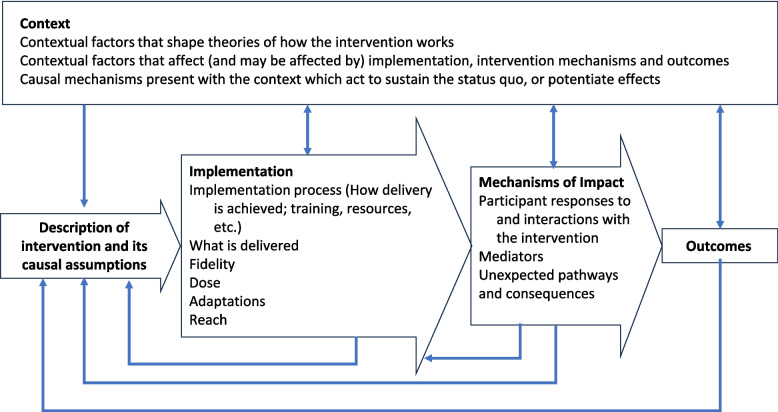
MRC guide to process evaluation(9)

Data was analysed using framework analysis [27]. Using the conceptual domains of RE-AIM (Table 2) and the MRC guide to process evaluation (Fig. 1), two independent researchers (BT, EW) constructed a thematic framework for analysis. Data analysis began with reading and achieving familiarity with the transcripts for preanalytical understanding. Next, the transcripts were coded line-by-line deductively and inductively to ensure no important aspect of the data was missed. A deductive approach was adopted to guide the initial phase of data analysis by efficiently assigning emergent codes to the thematic framework’s pre-established categories. Familiarization and coding were repeated iteratively to refine existing codes and emerging categories as definitions and meanings became clearer and more precise. Triangulation between coders, qualitative and musculoskeletal experts was performed to bolster rigor and establish validity by identifying areas of divergence, and uncover deeper meanings from inconsistencies in the codes, categories, and emergent themes. The data was reduced through a matrix comparing categories of data [27] and subsequently organized into the thematic framework. Data were sampled and analysed iteratively at regular intervals until saturation in themes was achieved. Microsoft Excel was used to analyze and manage the data.

##### Human ethics and consent to participate

Ethics approval (NHG DSRB ref no: 2020/00067; clinical trial number: not applicable) was obtained for this study. Informed consent to participate was obtained from each patient prior to data collection. This study was carried out in compliance with the Declaration of Helsinki.

## Results

One hundred and ten patients (55 control and 55 intervention) participated in the CONNACT RCT. Of the RCT’s 55 intervention patients, 22 were purposefully sampled for this process evaluation, no patient declined to participate. Table 3 summarizes the characteristics of the 22 patients. Table 4 summarized the characteristics of the 14 healthcare professionals (five physiotherapists, four orthopaedic surgeons, one rheumatologist, one psychologist, one medical social worker and two dieticians) also participated. Significant themes that emerged from the interviews are structured based on the RE-AIM framework (Reach, Effectiveness, Adoption, Implementation and Maintenance). Sub-themes and quotes amounting to their respective pre-established overarching themes based on RE-AIM are presented in Supplementary Table (see Additional File 4).

**Table 3:** Baseline characteristics of patients interviewed

No	Age & Gender	Language	Employment Status	KL Grade	KOOS_4_ (B – baseline, E – end point)	Improved/Deteriorated/Minimal change*	Time since Intervention completion (months)
1	65, F	English	Full-time [Manager]	2	46.98 (B) 88.03 (E)	Improved	4
2	68, F	English	Full-time [Catering assistant]	3	50.32 (B) 82.32 (E)	Improved	0
3	64, F	Mandarin	Retired	3	59.86 (B) 72.9 (E)	Improved	0
4	68, M	English	Part-time [Security officer]	4	44.12 (B) 53.8 (E)	Minimal Change	8
5	60, F	English	Full-time [Project planner]	2	54.3 (B) 75.33 (E)	Improved	8
6	64, F	Mandarin	Homemaker	4	64.78 (B) 61.11 (E)	Deteriorated	0
7	68, M	English	Retired	4	70.62 (B) 88.77 (E)	Improved	12
8	72, F	Mandarin	Homemaker	3	41.3 (B) 53.26 (E)	Improved	6
9	77, M	Mandarin	Retired	4	65.12 (B) 82.53 (E)	Improved	10
10	70, F	Mandarin	Homemaker	4	72.48 (B) 80.93(E)	Minimal Change	9
11	70, F	English	Retired	3	68.07 (B) 79.53 (E)	Improved	0
12	64, M	English	Full-time [Manager]	2	43.45 (B) 100 (E)	Improved	7
13	72, F	English	Retired	2	66.98 (B) 88.02 (E)	Improved	11
14	81, F	English	Retired	3	56.33 (B) 82.14 (E)	Improved	8
15	74, F	English	Part-time [Antenatal trainer]	4	63.72 (B) 80.24 (E)	Improved	11
16	72, M	English	Retired	4	55.56 (B) 57.99 (E)	Minimal Change	6
17	77, F	Mandarin	Retired	4	55.54 (B) 56.09 (E)	Minimal Change	0
18	69, M	English	Part-time [Quality surveyor]	3	59.47 (B) 85.49 (E)	Improved	0
19	66, M	English	Retired	3	43.35 (B) 35.82 (E)	Deteriorated	7
20	58, F	English	Homemaker	3	38.63 (B) 80.36 (E)	Improved	9
21	50, F	Mandarin	Homemaker	3	51.56 (B) 71.24 (E)	Improved	0
22	74, M	Mandarin	Retired	3	71.98 (B) 82.04 (E)	Improved	0
Gender		Language	Employment	KL Grade	KOOS_4_	Change	Time (months)
Males: *n* = 8 (36.4%)		English: *n* = 14 (63.6%)	Part-time *n* = 3 (13.6%)	KL 2 *n* = 4 (18.1%)	Baseline (B) Avg.: 56.57 SD.: 10.60	Minimal Change *n* = 4 (18.2%)	Avg.: 5.3 SD: 4.4
Females: *n* = 14 (63.6%)		Mandarin: *n* = 8 (36.4%)	Full-time *n* = 4 (18.2%) Homemaker *n* = 5 (22.7%)	KL 3 *n* = 10 (45.5%)	Endpoint (E) Avg.: 74.45 SD.: 15.27	Improved *n* = 16 (72.7%)	
Age Avg.: 68.3 SD: 6.92			Retired *n* = 10 (45.5%)	KL 4 *n* = 8 (36.4%)		Deteriorated *n* = 2 (9.1%)	

^*^ Minimal change defined as within 10 points of KOOS_4_ (minimally clinical importance difference [26])

**Table 4:** Characteristics of healthcare professionals interviewed

Subject		Gender	Involvement in intervention
PSY01	Principal psychologist	F	Yes
MSW01	Medical social worker	F	Yes
Diet01	Senior dietician	F	Yes
Diet02	Dietician	F	Yes
PT01	Principal physiotherapist	F	No
PT02	Principal physiotherapist	F	No
PT03	Principal physiotherapist	M	No
PT04	Physiotherapist	M	No
PT05	Senior physiotherapist	M	No
Doc01	Consultant (orthopedic)	M	No
Doc02	Consultant (orthopedic)	M	No
Doc03	Consultant (orthopedic)	M	No
Doc04	Consultant (orthopedic)	M	No
Doc05	Senior consultant (rheumatology)	F	No
	Psychologist: *n* = 1 (4.5%) Medical social worker: *n* = 1 (4.5%) Dietician: *n* = 2 (9.1%) Physiotherapist: *n* = 5 (22.7%) Doctors: n = 5 (22.7%)	Male *n* = 3 (13.6%) Female *n* = 3 (13.6%)	Yes: *n* = 4 (18.2%) No: *n* = 18 (81.8%)

### Reach: participation motivated by desire to avoid surgery, family/work commitments most common reason to decline participation

Patients with a suspected diagnosis of knee OA were referred by primary healthcare or emergency medicine doctor to the outpatient clinic at Tan Tock Seng Hospital’s Department of Orthopedic Surgery. A total of 287 eligible patients were invited to join the study, with 110 patients (38.3%) eventually enrolling and randomised. Of the 177 patients who were not enrolled, 44.6% (79/177) did not meet the inclusion criteria. Of the 55.4% (98/177) who met the inclusion criteria, 39.7% (39/98) declined to join the trial because they were unable to commit due to work or caregiving commitments and 19.4% (19/98) were disinterested in physiotherapy or other allied health interventions. Reasons for declining were captured in the recruitment log and are summarised in Table 5.

**Table 5:** recruitment logs with reasons for non-participation

Reasons	n (%)
Total patients invited, n(%)	287 (100)
Enrolled in main RCT and randomized	110 (38.3)
Not enrolled	177 (61.7)
Did not meet inclusion criteria	79 (27.5)
Does not meet KOOS_4_ score criteria	23 (8.0)
Language (Non-English or Chinese speaking)	10 (3.5)
Planned for knee surgery	3 (1.0)
Wheelchair bound	3 (1.0)
Does not meet KL criteria	1 (0.3)
Other medical condition	39 (13.6)
Concurrent MSK co-rmobidity	27 (9.4)
Cognitive/Psychological co-morbidity	2 (0.7)
Neurological co-morbidity	2 (0.7)
Other co-morbidity	8 (2.8)
Declined physiotherapy and other allied health intervention	19 (6.6)
Unable to commit due to work/caregiving commitments	39 (13.6)
Financial issue	1 (0.3)
Location of intervention not convenient	3 (1.0)
Other reasons	36 (12.5)

*KL* Kellgren Lawrence, *MSK* Musculoskeletal

A prominent theme for participation emerged from the qualitative data, including a deep desire to avoid surgery “*I was very happy because I don’t need to go operation…. I prepared for the worst, but if someone tell you, you don’t have to go through the worst, you can just do ABC, you get the same solution”* (Patient 20), *“The one that made me motivate me to join this program is the program itself, that you can avoid surgery by just exercise and reduce weight”* (Patient 18) with a less or non-invasive alternative that was perceived to be equally effective or on the recommendation of the managing physician where “*if the doctor recommends me to go, I will go, but I wouldn’t go on my own.”* (Patient 10)*.*

### Effectiveness

The RCT demonstrated that improvement for primary outcome KOOS_4_ over 12 months showed no significant difference (diff −0.12 [−5.59, 5.34]) between the control (hospital-based care) and intervention CONNACT. Considering the secondary outcome measures, the CONNACT intervention group demonstrated superior results compared to the control in physical performance, knee satisfaction, global perceived effect, and positive dietary change particularly over the short term. Table 6 provides a summary of the CONNACT RCT’s effectiveness results (see [7] for details on measurements and analysis of outcomes). Details on the analysis can be found in the main RCT paper [8].

**Table 6:** Summary of the CONNACT RCT results [7]

	**Descriptive statistics**^**#**^		**Main effect of group**^**a**^	
	**Usual Care**	**CONNACT**	**Difference (95% CI)**	**SE**
**Primary Outcome** [28] (12 month)				
KOOS_4_ score, mean ± SD	78.55 ± 16.15^#^	76.21 ± 14.83 ^#^	−1.86 (−9.11, 5.38)	2.9003
**Secondary Outcomes** (12 month)				
KOOS Pain, mean ± SD	83.82 ± 15.50 ^#^	80.84 ± 17.05 ^#^	−2.72 (−10.92, 5.48)	3.2832
KOOS Activities of Daily Living (ADL), mean ± SD	83.97 ± 16.99 ^#^	84.33 ± 14.03 ^#^	0.87 (−7.14, 8.87)	3.2048
KOOS Symptoms, mean ± SD	82.64 ± 16.58 ^#^	77.64 ± 17.25 ^#^	−4.04 (−12.75, 4.67)	3.4869
KOOS Quality of Life (QoL), mean ± SD	63.78 ± 24.98 ^#^	62.05 ± 20.82 ^#^	−1.76 (−12.17, 8.64)	4.1654
***Physical function outcomes*** [29]				
40 m gait speed, mean ± SD	1.26 ± 0.33^#^	1.40 ± 0.38 ^#^	0.11 (−0.07, 0.30)	0.0736
Time-up and go (TUG), mean ± SD	11.46 ± 3.55^#^	10.96 ± 3.61^#^	−0.48 (−2.59, 1. 62)	0.8439
Chair stand test (CST) 30 s reps, median (IQR)	11 (9, 13) ^#^	11 (9, 14) ^#^	0.02 (−0.19, 0.23)	0.0845
Stair-climb test (SCT), mean ± SD	9.79 ± 4.57 ^#^	8.32 ± 3.35 ^#^	−1.96 (−4.67, 0.75)	1.0858
UCLA [30], mean ± SD (measures physical activity)	4.59 ± 1.35 ^#^	4.45 ± 1.21	−0.10 (−0.82, 0.61)	0.2857
***Psychological outcomes***				
Pain Interference Scale (PEG) score [31], mean ± SD (measures pain intensity, interference with enjoyment of life, and interference with general activity)	2.48 ± 2.56 ^#^	1.94 ± 1.84 ^#^	−0.48 (−1.63, 0.68)	0.4616
Patient Health Questionnaire-4 (PHQ-4) [31], mean ± SD (measures anxiety and depression)	1.14 ± 1.71 ^#^	1.00 ± 1.58 ^#^	−0.06 (−1.31, 1.19)	0.5023
Patient Activated Measure (PAM) [32] High (≥ 3), n (%) (measures patient activation in managing health)	23 (76.7)	29 (87.9)	1.04 (−1.36, 345)	0.9622
***Dietetics outcomes***				
Body Mass Index (BMI), mean ± SD	26.62 ± 5.14	25.58 ± 3.81	−0.46 (−2.64, 1.73)	0.8741
***General outcomes***				
EQ-5D index [33], mean ± SD (measures quality of life)	0.78 ± 0.31^#^	0.78 ± 0.26^#^	0.00 (−0.12, 0.13)	0.0509
EQ-5D VAS [33], mean ± SD (measures quality of life)	79.27 ± 14.30 ^#^	78.64 ± 16.25	−0.27 (−7.92, 7.41)	3.0697
Cumulative Analgesia Consumption Scale (CACS) [34], mean ± SD (measures amount and potency of analgesia over one week)	0.24 ± 1.07 ^#^	0.24 ± 0.66	0.12 (−1.09, 1.33)	0.4851
Global Perceived Effect (GPE) [35], median (IQR) (measures patient current knee condition after treatment)	2.0 (1.5, 3.0)	2.0 (1.0, 3.0)	–0.86 (−2.22, 0.50)	0.5684
Patient Acceptable Symptom State (PASS) [36], n (%) (measures patient satisfaction with current knee function)	31 (72.1)	33 (78.6)	0.54 (−1.22, 2.31)	0.7357

^a^Bonferroni corrected estimated with 95% confidence interval

^#^Significant changes (*p* < 0.05) from baseline to 12 months (effect of time)

^*^p < 0.05

### Adoption: necessity, viability, and challenges of long-term sustainability

There was a perceived need for such an intervention in the community among the healthcare professionals *“I feel that a lot of these cases should be ideally managed in the community…so that they don’t get recurrent flare-ups that are so debilitating they feel they need to go to the hospital…. patients can be effectively managed in a group setting out in the community, so I think that’s a good initiative”* (PT04).

However, there were several concerns, and suggestions were put forward for the long-term sustainability of the intervention. First, in terms of the delivery format, the importance of tailoring to the individual was a key concern. Engaging the patients through interaction delivery strategies while keeping the exercise and educational material practical and relatable with use of interactive activities to the patient was highlighted several times. There was a concern that mixing languages within the same group class would be suboptimal and healthcare professionals running the class needed to empower patients rather than simply delivering didactic content. The expressed concerns were encapsulated by the following quote: “*The material is developed in English, so when we deliver it in Chinese, we might not be making it fun or relatable enough. Chinese is not my first language… and a lot of them just look at the screen… I think it’s very much a lecture, like a teaching session. For social workers, we do run therapeutic groups where we try to draw the answers from the group… From where I come from, we are all equal and I am there to facilitate to find an answer to your problems*” (MSW01).

Second, the viability of technology to complement intervention. While the benefit to technology was clearly recognized with *“I think it will be very helpful. You can argue that it may not be helpful across all, but if you can help a majority of them then I think it is effective enough.”* (Doc03) but concerns still exist around the tech savviness of the elderly as “*the elderlies that we interact with, they are not very familiar with technology. And having a smartphone is a luxury for most. Most of them use prepaid cards, and they need to learn how to set up and connect to Wi-fi, and they may not be using their own phone… not everyone has family members or people to teach them*.” (MSW01).

One highlighted the incompatibility of technology with socially complex older adults whereby “*The ones that are not workable are the 80 plus years old and the social isolated. They have no resources, no phone, nothing… This group requires a completely different approach cos they might not even want to get out of the house*” (PT01). Despite the promise of technology, the importance of physical interaction was still highlighted, “*Technology, no matter what, cannot be compared. When we communicate with others, we have our tone, mannerisms, even charisma: you can feel it. It cannot be compared to physical interactions and its effects, definitely not comparable*.” (Patient 03).

#### Adoption: suggestions for consideration

Interestingly, one felt *“We don’t have to segregate the classes… While they are doing exercises, are we able to teach them a bit about psychology? Are we able to talk to them a little bit about diet? Maybe the therapists might need to learn a little bit about the psychology, nutrition… wouldn’t it be more efficient if everything is incorporated in one session”* (MSW01)*.* This means transiting from a multidisciplinary to a transdisciplinary approach where a single person with multiple skill sets delivers the intervention independently. This would shift the burden away from a team of hospital-based specialists and allied health professionals towards a more resource efficient team of well-trained, all-rounded community-based health workers.

A few healthcare professionals advocated the support for peer leaders or expert patients in line with greater patient empowerment *“There has to be a leader within the group to motivate others. It has to come from the group itself. That’s the only way the system is sustainable”* (PT01). A patient concurred “*whenever there is a class, I believe you need to have a leader… I feel that one of you all should take the lead, because we are all strangers*” (Patient 18). These peer leaders could encourage and support post-program activities within the groups in a patient-led fashion.

The importance of post-program transitioning coupled with refresher or booster sessions was suggested *“Actually unless you go for a refresher course…. after a few months, you almost forget all of them, almost 80–90% already forgotten. So, maybe refresher course can help to bring back the memory”* (Patient 12) to support long term maintenance of the behavioural modifications that were achieved during the 12-week intervention.

### Implementation

There were two key themes (context and mechanism of impact) in the implementation domain. Context includes anything external to the intervention that may act as a barrier or facilitator to its implementation. The mechanism of impact seeks to identify the potential causal pathways that resulted in the changes seen.

#### Context: two ends of the spectrum on pre-existing mindsets and expectations, majority with passive acceptance and resignation to condition

On one end of the spectrum, there were patients “*that will have high expectations: wanting to go back (to normalcy), to be as strong and fit as before, and it may be due to their role in the family or society. Maybe they used to be high fliers, used to be very active, play sports…and that loss of function then matches the loss of status”* (PSY01)*.* This group demanded a quick fix solution that was often accompanied by an unhelpful pain belief and “*perception of pain is"you need to push through pain","pain is good", and they keep aggravating the situation, they don’t know when to stop”* (PT03) leading them to constantly push through the pain. This need for quick resolutions of symptoms stemmed from reasons like treatment cost with *“many patients are breadwinners or blue-collar workers, whereby if they don’t work, they don’t have money… for them, they need a quick solution because they need to get on their feet again to earn money… Exercise is a luxury”* (MSW01) or not wanting to be a burden to their family “*I need my children to bring me here, but my children working”* (PT02).

On the other end of the spectrum, most patients exhibited passive acceptance of their condition having *“this misconception that ageing and weakness come together”* (PT03)*.* This belief was often reinforced by family, friends and colleagues with “*they* (colleagues and family) *say this is natural ageing process, so it seems like unavoidable when we reach certain age this knee problem will occur somehow. So just take it at face value… I believe probably is due to wear and tear, so I have to live with this problem*.” (Patient 12). “*Some elderly people have accepted that they won’t be as mobile”* (Doc05), not wanting or believing anything could be done with a “*passive acceptance that says,"oh, I just take it as part of old age, so I just bear with the pain", and they just continue to do things normally….it’s part of old age, I can’t do anything about it so I just go on with life as per normal”* (PSY01)*.* Many patients felt that it is the doctor’s job to resolve their symptoms where* “older patients who have that expectation of doctors:"it’s the doctor’s job to fix things, not mine","I don’t get involved with my management". It’s a fairly passive concept or attitude to their disease, or lack of knowledge, or lack of interest…"doctor is the one who knows everything and does everything, and I don’t do anything","what the doctor tells me, I’ll just do it, but I expect the doctor to fix it"* (Doc05)*.* Traditional Chinese Medicine (TCM) for temporary pain relief emerged as a strong theme but in differing viewpoints with many patients feeling that it is “*part of…..culture, they feel Chinese medicine is centuries old, and it’s safe and not poisonous”* (Doc05) but others feeling that “*I just don’t believe in TCM… I don’t know if they* (TCM physicians) *gone through any course like a doctor who has gone through medical school… TCM to me, it's just like taking herbs and this and that, not proven, so I don’t really go.*” (Patient 14).

#### Mechanism of impact: active acceptance and self-efficacy, group class support, intervention synergy

Within the larger theme of mechanism of impact, there were 3 key subthemes that helped us understand and interpret both the positive and negative results that were observed in the RCT.

##### Active acceptance and self-efficacy

For several patients, there was a shift in patients’ mindsets towards active acceptance and self-efficacy over the course of the intervention. The PSY and EDU elements of the intervention were grounded on the acceptance and commitment therapy (ACT) approach, where in contrast to passive acceptance, patients were not only encouraged to accept their limitations but also to make an active commitment to take dedicated steps to improve. A key enabler for this was a strong focus throughout the intervention on patient education and empowerment. Through education and empowerment, patients learnt not only the “why” of knee OA but also the “what” and “how”, and were encouraged to take tangible steps to better cope with their condition. The following quote encapsulated this key mindset shift. Unfortunately, not all patients interviewed exhibited this mindset change.

“This programme helped me to not only physically with the exercising, relieving the pain, but more importantly, positive thinking. I have to be in the present, whereas I used to push everything back, ignore, I have to confront it…. I have to live with it, but I know how to deal with it…….carry on as normally as I can… You cannot just sit back and suffer the pain and then wait for it to get better, not going to work that way. You have to take responsibility yourself…. because I accepted my condition because I know I can do something about it when the pain comes…. I am in control, I don’t worry so much about it, it doesn’t make me so helpless…. with that kind of positive thinking, it helps me to carry on” (Patient 14).

##### Group class support and inherent tension

The benefits of a group class were also highlighted. Many patients pointed out how a group class gave them a sense that they were not alone in their journey “*because you're in a class, you realise"hey I'm not alone, I am normal, other people also experiencing the same thing as me”* (Patient 20) and gradually became a source of support, motivation and to a certain extent, peer pressure as they underwent the intervention together, learning not only from the facilitators but from each other. Patients felt that “*By conducting the programme in groups, we can discuss among ourselves and this helps us to learn and understand more… The group session in this programme serves as a form of support, and everyone can share, so that you don’t feel lonely when you encounter issues… it's a form of encouragement, and it gives us something to look forward to*.” (Patient 06). These positive experiences encouraged adherence and strong maintenance throughout the intervention period, “When I feel like dropping off, and I think about the group, and I should do it, because for the group’s sake… we sort of encourage one another."Oh my goal is to walk this distance", and then just carry on with what they are learning from this programme"(Patient 14). However, the inherent tension and limitation of a group class was also raised. Several patients expressed a preference for personal treatment, pointing out a lack of flexibility in the group class and a lack of personalization with sentiments like *“I was the oldest there. Some activities and exercises parts are quite difficult… About two or three that I couldn’t do”* (Patient 17).

##### Intervention synergy

The third subtheme that emerged was the holistic and synergistic nature of the CONNACT intervention. Each element brought about its own benefit. The physiotherapy class was foundational for the intervention through exercise and physical activity. As patients were getting stronger through gradual progression of exercise intensity over the 12 weeks, patients were able to gain confidence in what they were able to achieve. The PSY and EDU classes helped bring about positive mindset changes that were elaborated in the first theme. The NND classes helped empower patients by improving their dietary knowledge, highlighting the fact that weight loss plays in improving pain and function and giving patients practical steps to change their diet. Most importantly, all the elements were able to work synergistically together, complementing each other and some patients were able to appreciate that sharing that *“All the three approaches… they complement one another… they are all equally helpful. The exercises are helpful in reducing the pain, the psychology help me to mentally, emotionally accept my pain, the nutrition help me weight loss… if I had not attended either one of the sessions, it would have been incomplete”* (Patient 14)*.*

#### Maintenance: incorporating exercise into regular routines, perceived lack of time and inertia barriers

Patients highlighted the importance of incorporating exercise and physical activity into their regular daily activities recognizing the need to *“change your habit… you have to be consistent… if you are not consistent, then it would not be able to help you improve. So, we learn to be consistent and what to do when you got this problem…. everybody is the same, no time, so must make time to do”* (Patient 01) and some as part of a community-based program such as a walking group or Taichi class *“some of the exercises that we were taught in this programme, is also inside this Qi Gong that I do every day. So, when I do my Qi Gong, I remember what I learn here, and when I stretch, I really stretch”* (Patient 20). Seeing improvements though the course of the intervention gave the patients encouragement to continue preserving with their exercises as they strived towards greater improvement and functional recovery with patients reporting that *“I feel there's a progressive healing, progressive. It's gradual, after three months I think my pain really subside quite a certain extent*” (Patient 12) and *“With more exercises, and you see a bit of your weight going down, the motivation is there… I have less problem of the excruciating pain than when I first started”* (Patient 15)*.*

However, there was significant barriers identified as well which included a perceived lack of time and inertia where patients shared that “*I have to admit partly is because of my own fault in the sense that I wasn’t so diligent in doing the exercises… I have to be honest, most of the time is just being lazy… sometimes you’re watching (TV), you can sit down for two hours… I wish I were more diligent*” (Patient 13) and complacency when symptoms improve when “*, I almost forget about it because there's no pain, seems like there's no motivation to do the exercises*” (Patient 12) and *I sort of gradually fell back and didn’t bother with the exercises because the pain was not bothering me so much, I could put up with it*” (Patient 14).

## Discussion

This study examined the implementation of a community based multidisciplinary intervention (CONNACT) by identifying key themes of context (pre-existing health belief and treatment expectation), mechanism of impact (active acceptance and self-efficacy, education and empowerment, group class support, intervention synergy) and maintenance (incorporation into regular routines, community programmes and celebrating small wins) to understand and interpret the RCT outcomes through an explanatory sequential mixed methods approach. Identifying barriers coupled with proposed changes for long term sustainability (tailoring to the individual, transdisciplinary approach, peer leaders, embracing technology and post-program transition through booster or refresher sessions) will help to inform the next phase of wider, streamlined, and more effacious implementation within the community.

### [Reach] reaching out to the knee OA population: overcoming resistance to participation

Addressing the factors that impact the reach of the CONNACT intervention would be key to successfully implementing this intervention on a larger scale. Of all the patients who met the inclusion criteria, only 53% participated in the trial. The top reasons identified from the quantitative recruitment logs were difficulty with time commitment for a full 12-week intervention due to work or family, a general disinterest in physiotherapy, and unfavourable intervention logistics (location, timing etc.). The interviews also revealed the lack of belief in exercise was in alignment with unhelpful pre-existing beliefs that were identified in the context domain. In contrast, a desire to avoid surgery appeared to be a key reason why patients were keen to join the intervention.

Moving forwarded for implementation, understanding the reasons why patient join or decline such interventions, including addressing underlying health beliefs, treatment expectations and misconceptions in the context of local cultural practices such as TCM are critical when counselling patients in order to reach as much of the target population with knee OA. In addition, addressing logistics-related (location, timing) issues through provision of weekend or evening classes and flexible class schedules are key to reaching patients with pre-existing work or family commitments.

### Effectiveness, maintenance, implementation] evolving from multidisciplinary to transdisciplinary approach to enhance 3e (exercise, education, empowerment) synergy

Interviews indicated a key benefit of CONNACT was the development of positive mindset changes and active acceptance in patients supported by the synergy among its exercise, education, and patient empowerment components. This was congruent with CONNACT patients outperforming the control group in global perceived effect and symptom satisfaction [7]. The combination of positive mindset, better perceived global improvement, and satisfaction with symptoms, allowed patients to “embrace” exercise as part of their daily activities, have the confidence to “deal with it”, and make and maintain necessary lifestyle modifications. In addition, experiencing the benefits (e.g. improved strength, pain and energy levels) and being able to adapt exercise and physical activity according to their needs and limitations (e.g. time, energy, physical function, etc.) could have led these patients to develop a result-oriented mindset about exercise and physical activity. This differs from Boswell et al.’s finding of knee OA individuals possessing a higher appeal-focused mindset (e.g. fun, social, and indulgent) about physical activity [37]. Unfortunately, despite its best efforts, the multidisciplinary CONNACT could not alter deep-seated, pre-existing unhelpful health beliefs and passive attitudes, which prevented positive mindset changes and impacting its overall outcome. This is congruent with Mathieu et al. who stated unhelpful thoughts are a barrier to effective psychological management, and suggested incorporating the perspectives of OA patients in the development of education materials [38].

Progressing from a multidisciplinary to a transdisciplinary design is a solution suggested by the healthcare professionals. Transdisciplinary practice has become increasingly prevalent in healthcare due to its ability to economically improve the effectiveness and efficiency of healthcare via streamlined utilization of resources as care is provided by a smaller, cross-trained team [39] compared to a larger, specialised multidisciplinary team. This transdisciplinary care can be delivered by traditional healthcare professionals such as doctors, nurses, allied health professionals, and even by trained lay people like health coaches. A systematic review and metanalysis has shown that health coaching reduces both disability and pain in people with chronic musculoskeletal conditions like knee OA [40].

### Implementation] optimizing care delivery model

#### *Greater* personalization and optimization of exercise component

Congruent with literature [41, 42], this study strongly indicated patient education and psychological interventions need to be conducted in tandem with an effective exercise intervention as part of a comprehensive multidisciplinary intervention to demonstrate their fullest effects and benefits. Although CONNACT’s eight exercise sessions showed some short to mid-term positive results in the physical performance measures, the effects quickly petered out after one year. Similar interventions that showed long-term effectiveness had significantly longer exercise components that last up to 18 months with dedicated post-program booster sessions [43–45]. Hence, in the next phase of its wider implementation within the community, CONNACT’s exercise component may be intensified (i.e. duration per session and number of sessions) according to the patient’s needs without unnecessarily straining their resources (e.g. time, energy, finances, motivation, etc.).

#### Personalized group-based delivery

Unlike Allen et al. who found no differences in the outcomes between group and individual therapy for knee OA [46], the CONNACT patients’ mixed experiences towards the group-based delivery format highlighted implementation challenges that potentially resulted in the similarly mixed outcomes observed in the RCT. On the one hand, patients enjoyed the socialization aspect of group therapy knowing that they were not alone, supported by fellow patients and, to a certain extent, motivated by positive peer pressure to exercise and make lifestyle modifications. On other hand, patients pointed out the lack of logistic flexibility (i.e. class dates and times) and the lack of personalization in their treatment. These, together with the RCT’s results, are congruent with a systematic review on the effectiveness of individual and group-based exercise therapy for musculoskeletal conditions reported small, clinically insignificant differences in pain and functional outcomes, but concluded that group interventions may be preferable, given their similar effectiveness and potentially lower healthcare costs [47]. Nevertheless, the community, group-based, multi-disciplinary CONNACT intervention fulfils Mathieu’s suggestion of enabling people with OA, who recognised the significance of functional, psychological, and social self-management skills, to learn ways to deal with the consequences of OA and pain from others like themselves [38].

Moving forward, the CONNACT intervention would strongly consider options to flexibly cater to both individual and group sessions to optimize its patients’ outcomes. The individual sessions would be more suited for very high or very low functioning patients who would perform specifically prescribed exercises that address their needs under close supervision. Whereas the group sessions would need to take a step further to personalized yet optimally balanced treatment for patients. For example, a qualitative study investigating the individualization of group exercises for multiple sclerosis patients identified several key factors such as initial individual assessment, equally distributed attention and engagement, targeted exercises with one-to-one adjustments for exercises tailored to the patient’s specific symptoms and needs, and positive verbalization of improvements [48].

### Strengths and limitations

A robust methodology was employed for this process evaluation using the RE-AIM framework, a well-established framework that has been used specifically within the context of knee OA [49]. The qualitative interviews for the process evaluation were designed based on the MRC guide to process evaluation [15], covering the key domains such as context, implementation, and mechanism of impact.

There are several limitations to the study. Firstly, while an effort was made to measure compliance at a patient level, intervention delivery fidelity was not measured. The intervention was delivered by a small core group of clinicians who were part of the intervention design team; hence, the need to measure fidelity was deemed unnecessary for this study. Secondly, the COVID epidemic impacted the ability of patients to gather in groups to exercise post-program, which might have impacted long term exercise compliance rates. Thirdly, the healthcare professionals who were interviewed were either directly or indirectly involved in CONNACT. This might not have demonstrated a true reflection of the adoption of such an intervention by the general medical community, key decision makers and administrators. This study, conducted in a highly-developed multicultural and multi-racial Southeast Asian society, provided sociocultural insights into contextual factors influencing the effectiveness and implementation of the community-based CONNACT intervention. Due to differences in sociocultural contexts and challenges, the study’s findings are mostly unique to Singapore’s sociocultural context and cannot be generalized to all Asian societies.

## Conclusion

This process evaluation used the RE-AIM framework and MRC guide to process evaluation to qualitatively examine the implementation of the CONNACT RCT, a community-based multidisciplinary intervention. Themes such as understanding barriers to outreach, promoting positive mindset change through education and empowerment, personalization within a group setting and incorporating a transdisciplinary approach and expert patients will inform the next phase of implementation. In future, CONNACT and similar programs should consider investigating and addressing the factors for declining participation (e.g. health beliefs, treatment expectations, misconceptions, logistical inconveniences, etc.) to increase their reach and participation rates. To improve its effectiveness and treatment experience and satisfaction, the group sessions could integrate components such as initial individual assessment, equally distributed attention, targeted individually-tailored exercises, and praises of improvements [48]. Although the exercise component may be intensified based on the patient’s needs, it should not unnecessarily strain the patient’s resources (e.g. time, energy, finances, motivation, etc.). Finally, evolving from a complex resource-intensive multidisciplinary intervention to a streamlined resource-efficient transdisciplinary intervention could facilitate its long-term maintenance.

## Supplementary Information

Supplementary Material 1.

Supplementary Material 2.

Supplementary Material 3.

Supplementary Material 4.

## Data Availability

Data is provided within the manuscript as quotes and in Supplementary Table (additional file 4). The de-identified datasets used and/or analysed during the current study are available from the corresponding author on reasonable request.
