# Supplementary material for: Collaborative model of care between orthopaedics and allied health professionals in knee osteoarthritis (CONNACT): process evaluation of an effectiveness-implementation hybrid randomized control trial
Source: BMC Musculoskelet Disord. 2025 Sep 30;26:876. doi: 10.1186/s12891-025-08925-0 (PMC12487322; doi:10.1186/s12891-025-08925-0)
Supplement: Supplementary file 2 — Supplementary Material 2. [file 12891_2025_8925_MOESM2_ESM.docx]

| **OFFICIAL USE ONLY** | |
| --- | --- |
| **Doc Name :** Intervention patient interview topic guide (with prompts) | |
| **Doc Number : 1.0** | |
| **Doc Version : 2.0** | **Date : 12/03/2020** |

**Intervention Patient Interview Topic Guide (with prompts)**

1. Patient’s Current Condition

- How are you feeling today?

1. Patient’s Initial Beliefs and Attitude

Please share with me some **thoughts and feelings** about your **knee condition before treatment**.

(Beliefs of cause, aggravation factors, pain, cure/relief)

- How did it **affect** you?

(everyday life: mentally, socially, emotionally, financially)

- What have **others said** / **advised** about your knee condition?

(Impact on feelings, thoughts, action, behaviour, etc)

1. Patient Treatment Process, Experience, and Decision-making

Please share with me **some things you have done** to **manage** your **knee pain** before the treatment program.

(Activities, Strategies, Adaptations: effectiveness, how easy, challenges)

- What made you **decide** to **(not) seek** medical **help**?

(Level of pain, personal reasons, worsening condition, family pressure, etc)

- How did you **seek treatment information**?

(Source of information? How well-informed? Understanding of information?)

- What **issues** did you **face** when **trying to seek treatment**?
- How **helpful** was the **treatment(s)** you had **tried**?

(e.g. TCM, etc.) (Effectiveness, cost, reliability, usefulness, complexity, etc)

- **What** did the **doctor recommend**?

(Thoughts, feelings, perceived difficulties, etc)

- What will make you **not consider** total knee replacement **surgery**?
- **How** do you think your **life will be after** the **surgery**?

1. Patient’s Evaluation of Treatment

Please share with me some **thoughts** about the **treatment** you have **received**.

- What made (or motivated) you to **attend the treatment**?

(Relieve pain, upfront payment, incentives, social interaction, pressure, goals, etc)

- **Which part** was **most helpful** and which was **least helpful**?
- Do you have any **suggestions** on how the **treatment** can be **improved**?
- Would you **benefit more** from **minimum ad hoc physiotherapy**?

1. Patient’s Experience of Group Classes and Healthcare Professionals

Please share your **experience** of the **group classes**.

- Did you **enjoy** / **benefit from** the **group classes**?
- Did you **benefit** from the **sharing** by the **expert patient**?

(Useful, motivating, reassuring, knowledgeable, etc.)

- Did your **healthcare providers** **meet** your **expectations**?

(Knowledge support, content delivery, listen and address concerns, etc.)

1. Patient’s Maintenance

**After the program**, how is your **knee compared to before**?

(Improve / Worsen: Function, flexibility, strength, pain, stiffness, flare frequency, etc)

- What do you think are the **reason(s)** for the **improvement/deterioration**?

(Incentives, knowledge, skills, motivation, personal strengths, support, issues, etc)

- Did the **program help** your **condition**?
- What are your **next steps** / **plans**?

Are there any **changes** that were **made** to your **lifestyle**?

(Physical activity, diet, mindset, learning / experimenting new things, etc)

- What **supported** / **prevented** you from **making changes**?

(Incentives, knowledge, skills, motivation, personal strengths, support, issues, etc)

- What are your **next steps** / **plans**?

1. Patient’s Preferences & Challenges

Would you prefer if someone **connects closely** with you, **reminds** you about your treatment schedule/follow-ups, **checks** on your health/progress, and **gives you encouragement/advice**?

Would you prefer **using technology,** in addition your treatment, to **manage your OA knee**?

(Fitness apps, wearables, tele-medicine: preferences, challenges, alternatives, etc.)

1. Patient’s Concluding Thoughts

Is there **anything else** you would like to tell us regarding your **knee condition** or **treatment**, that we **did not talk about**?
