# Supplementary material for: Collaborative model of care between orthopaedics and allied health professionals in knee osteoarthritis (CONNACT): process evaluation of an effectiveness-implementation hybrid randomized control trial
Source: BMC Musculoskelet Disord. 2025 Sep 30;26:876. doi: 10.1186/s12891-025-08925-0 (PMC12487322; doi:10.1186/s12891-025-08925-0)
Supplement: Supplementary file 3 — Supplementary Material 3. [file 12891_2025_8925_MOESM3_ESM.docx]

Introducing HCP

| Please tell me about **yourself**. | Age  Years of experience  Qualifications  Current position  **Frequency** of **treating** elderly with chronic (knee) pain  **How often** do you **work with** **other healthcare professionals** when treating chronic (knee) pain? | HCP’s experience and expertise |
| --- | --- | --- |

HCP’s Evaluation of Singapore’s Current Model of Care for Chronic (Knee) Pain in Elderly Patients

| Can you tell me about the **standard treatment** for chronic (knee) pain in elderly patients? |  | Understanding Singapore’s standard treatment of chronic (knee) pain in elderly patients |
| --- | --- | --- |
| What are your **views** on the **current local model of care** for chronic pain patients?  What are the **views** of your **colleagues**? | Can you describe the **support and facilities** provided by the hospital / centre to **help** **patients** manage their chronic (knee) pain?  **Strengths** /**issues**?  How are **patients affected**? | Model of care: Facilitators & barriers  Impact of MoC on patients |
| Based on your experience, **how informed are chronic pain patients** on their conditions? | How would **more information help**?  Would chronic (knee) pain **education campaign** be **beneficial**? | HCP’s perspective on patient’s information needs |

Healthcare Professional’s (HCP) Evaluation of Treatment

| Please tell me about your **experiences** of **delivering treatment** to elderly chronic (knee) pain patients. | Feelings about **role** / **contribution** in treatment  **Feelings** about treatment  **Interactions** with **elderly chronic pain patients** during treatment sessions | HCP’s experience and attitude towards treatment delivery  HCP’s experience of interacting with elderly chronic pain patients |
| --- | --- | --- |
| How are **patients reacting** to your sessions? | What is **preventing** patients from **improving** or **adhering** to treatment?  What can **help** them? | Patient’s reaction to model of care |
| Do you think chronic (knee) pain patients will **benefit** from **only** **individualised physiotherapy**? | **How effective** is this? | Efficacy of treatment |
| What is **preventing** you from **delivering better treatment** to patients? | If given the freedom, how would you have **done things differently**? | HCP’s concerns and needs in management of chronic pain |
| What **additional services** / **improvements** do you think are **needed** for the treatment to **provide better care**? | Education  Psychology  Nutrition and dietetics  Others |  |

How consistent is the delivery of content?

Healthcare Professional’s Perspectives on Upscaling Elements

| Presently, there is a multidisciplinary treatment program for treating knee OA in elderly patients. The program consists of Education, Psychology, Nutrition & Dietetics, and Physiotherapy components in small, group classes in a community setting. | | |
| --- | --- | --- |
| What are your opinions on using a **multidisciplinary approach** to tackle and improve knee OA? |  | HCP’s perspective on using multidisciplinary approach |
| How can the multidisciplinary treatment program **attract** more **healthcare professionals** like yourself to **be part of it**? | How can it **attract** **patients**? | HCP’s adoption of multidisciplinary program |

| In order to provide greater individualised treatment and assistance, a health partner (coach) will connect closely with elderly OA knee patients, reminds them about their treatment schedule and follow-ups, checks on their health and progress, and gives encouragement and advice. | | |
| --- | --- | --- |
| What are your thoughts on **assigning a partner (or coach)** to elderly knee OA patients as part of their treatment? | Characteristics?  Qualifications & Skills?  Effectiveness? | HCP’s perspective on health partner (coach) |
| What are your opinions on **integrating technology** into the **management of knee OA** for **elderly** patients? | Implementation issues? | HCP’s perspective on using technology |
| Based on your expertise, can patients be **incentivised** to **adhere** to **treatment**? | **Can** patients be **incentivised**?  W**hat form of incentive** will **appeal** and **work**? | HCP’s perspective on incentivisation |

Possible findings (Healthcare Professionals)

| Issues with Current Model-of-Care | 1. Long waiting list 2. Time constraints 3. Oversubscribed service 4. Patients do not understand the above issues |
| --- | --- |
| Factors Preventing Healthcare Professionals from Delivering Better Care | 1. Staff shortage 2. Overcrowding of patients 3. Heavy workload 4. Insufficient time with patients |
| Factors Preventing Patients from Improving or Adhering to Treatment | 1. Lack of education 2. Patients are unlikely to accept equipment-based exercises due to safety issues, economic situations, etc. 3. Different cultures may be physically more active than others (i.e. Chinese are physically more active than Malays) 4. Unmotivated – family, work reasons 5. Due to old age, sedentary lifestyle has become a norm 6. Even if patient does buy exercise equipment, their enthusiasm tapers off because the home environment might not be encouraging. |
| Factors Enabling Patients to Improve or Adhere to Treatment | 1. Influence of caregivers and educational level of family members can contribute to positive reinforcement 2. Social support increases engagement in treatment 3. Step-by-step illustrations are helpful 4. Comprehensive info will aid family members and caregivers in assisting patient 5. Exercises should be modified depending on the objective (endurance, strength, balance, flexibility) without compromising the intervention’s core components 6. Motivation is extremely important for good outcomes. 7. Group discussions, high education level and sharing sessions can improve motivation. 8. Highly motivated patients will in-turn motivate other patients 9. Patients might be more motivated outside (e.g. at the gym) then at home |
| Other Facilitating Factors | 1. Pre-assessment of patient’s background may help identify the best strategies for individualising treatment 2. Continuous engagement (during and after program) |
| Increasing Patient Uptake of Multidisciplinary Program | 1. Different ethnic groups, languages, beliefs and cultures – may influence implementation and acceptance of program. 2. Education may help people to understand the intervention. |

Source: Hasan (2019), unpublished PhD thesis

Adaptation to cultural contexts is defined as “the systematic modification of an evidence-based treatment or intervention protocol to consider language, culture, and context in such a way that it is compatible with the client's cultural patterns, meanings, and values” (Bernal et al., 2009).
