# Supplementary material for: Collaborative model of care between orthopaedics and allied health professionals in knee osteoarthritis (CONNACT): process evaluation of an effectiveness-implementation hybrid randomized control trial
Source: BMC Musculoskelet Disord. 2025 Sep 30;26:876. doi: 10.1186/s12891-025-08925-0 (PMC12487322; doi:10.1186/s12891-025-08925-0)
Supplement: Supplementary file 4 — Supplementary Material 4. [file 12891_2025_8925_MOESM4_ESM.docx]

| Domains and Themes | Sub-themes |
| --- | --- |
| RE-AIM domain: **Reach**  Perspective: **Patients**  Theme: **Participation motivated by desire to avoid surgery** | **Intrinsic and extrinsic motivations** (n = 10)  “*If it can reduce my pain, that is the biggest motivation*.” (P03)  “*If you want to get better, you have to go… If you are not going to turn up, why bother agreeing to go for it. It's your own pain, if you don’t go, you will just suffer… I don’t want to suffer. I have to go no matter what to heal myself.*” (P10)  “*Just tell myself I have to self-motivated to complete the course because this will bring a lot of benefit to me, so I have to motivate myself to attend the course, each session*.” (P12)  **Desire/intention to avoid surgery** (n = 12)  “*If still bearable, I don’t think I will go, unless really cannot... I will not consider.*” (P01)  “*If physiotherapy can improve my knee condition and reduce pain, then it would be good. It would be better not to do surgery*” (P06)  “*I don’t need a surgery to solve the pain problem… the new knee does not really give you perfect function, still have some problem… Such a marginal improvement, definitely no brainer, don’t need to go through that*.” (P20)  **Factors deterring surgery** (n = 12)  “*Surgery depends on your age because now I am 60+, if I have the surgery, maybe only can last me maximum 10 years, after that… I have to live through another suffering. I don’t want!*” (P02)  “*To me I have lots of things to do that I cannot undergo surgery. With surgery I cannot teach anymore, go out and join my friends for line dancing, do housework. Who is going to do for me?... you are confined for 3 months. To me that 3 months is precious. You can’t do much except exercise*” (P15)  “*Even if it continues like that, I still can move about... the other thing is, I’m not working, I don’t have an income. If I go for surgery, wah… it’s expensive! Even if I recover, how much can I do?*” (P16)  **Deference to doctor’s advice** (n = 5)  “*Frankly when I came to see I have no idea, I need the doctor to advise what am I to do. Because he will be in the best position to advise… I personally feel that for cases like this, you should not allow us to choose… I’m looking for you to help me. End of the day, we just got to take your instructions and follow… we might choose the wrong one because we don’t know what we are choosing*.” (P07)  “*I believe it is professional advice… furthermore of course previously I have also read about it before on my own… firstly, you would trust the doctor’s words*.” (P03) |
| RE-AIM domain: **Adoption**  Perspective: **Healthcare professionals**  Theme: **Need for and benefits of a community multi-disciplinary intervention** | **Need for community intervention** (n = 9)  “*I think it’s good, because it’s out there in the community... for patients' accessibility. With the accessibility of care and location, patient may be more compliant.*” (PT02)  “*A lot of simple cases could just be seen in the community or without a referral. But it is mostly red tapes… A simple problem could be made more complicated, or the pain could be worse by the time they see us… They would be either managing… worse by themselves.*” (PT03)  “*Most of the OA knee patients has… low complexity. If they can be managed in the community, then it doesn’t choke up the tertiary system down the road… The community is where they stay, more accessible, and if they are managed in the community, then it’ll be good!*” (PT04)  **Need for multi-disciplinary** **intervention** (n = 8)  “*A multi-D would help so that the patient don’t need to wait so long. In certain interventions… it would be good to do something like a multi-d or trans-d, so that would reduce the number of times the patient needs to see a healthcare professional.*” (Diet01)  “*The physio session was just really about physio, it’s got no element on diet or pain. You want to have every professional who is involved to be repeating your messages and reinforcing each other.*” (PT01)  “*You need a group of allied health professionals who specifically look after OA knee patients. Theoretically, if you have a clinic for patients with degeneration that has physiotherapist, psychologist, occupational therapist, nurse educator… a combined allied health service would be good.*” (Doc04) |
| RE-AIM domain: **Adoption**  Perspective: **Healthcare professionals**  Theme: **Effective lessons and interactive content** | **Hands-on/interactive activities** (n = 3)  “*Like cooking classes, how to tweak the recipes to make it healthier, if majority of patients were to cook at home... if it’s eating out, then it can be like maybe done in a hawker centre or something, so it will teach you on what to buy.*” (Diet02)  “*Before we introduce the healthy plate, everyone will pack or bring their lunch… they can bring their favourite drinks and snacks for a tea session. This is one way for me to gather information on the participants without asking them to do questionnaires… they cannot hide anything from me, they also don’t need to remember what they had eaten.*” (Diet01)  “*Let's say the lesson is about setting goals, are we able to bring the group out and demonstrate to them how it can be done? As in there will be not so much of sitting there and listening, but more engagement in activity… This would make it more relatable and at the same time we can tackle real difficulties that they face.*” (MSW01)  **Lesson delivery** (n = 4)  “*When we have to run the program in Mandarin, which for most of us Mandarin is not our first language… it is stressful in the sense that there’s certain expectations on both ends… my grasp of the language to be able to reframe some of the things they said, use very good examples and present that very accurately in Mandarin is the main barrier.*” (Psy01)  “*Technically, you change the way you deliver things like slow down a bit or repeat… the content we deliver is quite similar with what we have. But how much the patient can absorb depends on age… Whatever we teach, I didn’t really expect them to know.*” (Diet01)  “*Traditionally, dietician’s sessions tend to be more educational, lecture style… moving forward from there – what are you gonna do to bring about that change in behaviour to in order to achieve their actual goal?*” (Diet02) |
| RE-AIM domain: **Adoption (technological)**  Perspective: **Healthcare professionals**  Theme: **Viability of technology to complement intervention** | **Feasibility of technology for patients** (n = 7)  “*Things like gamification could be something interesting... maybe technology can come in if you can use AR... When they get excited, then it could work*.” (Psy01)  “*I think it will be very helpful. You can argue that it may not be helpful across all, but if you can help a majority of them then I think it is effective enough*.” (Doc03)  “*I think it’s a viable means. Often, just for a follow up to find out how they are managing, if something were to happen in the community, a flare of symptoms, or don’t know how to manage their conditions, then this remote access is a viable alternative and it’s something reassuring to patients*.” (PT04)  **Issues and limitations of technology** (n = 10)  “*If they’re Chinese-speaking, then that’s where the challenge comes in. Unless the app runs in mandarin function then it’ll be fine*.” (Diet02)  “*They* (i.e. patients) *may not know which one (i.e. apps) is the better or other. So, maybe there is some kind of guidance... But elderly, they don’t even press their phone, so it needs to be a bit more hands on*.” (Diet01)  “*Those* (patients) *living alone and not savvy. That's a double whammy, because you can launch this, but if the population is not aligned to technology, then you're not really not targeting the population that you want… then it's defeating the purpose*.” (PT05) |
| RE-AIM domain: **Adoption (technological)**  Perspective: **Patients**  Theme: **Receptive to technology but prefers physical interaction** | **Receptivity to technology** (n = 17)  “*Although this sort of things you can google and find out, but it’s good to have a dedicated app for the purpose. I think that might be a good idea.*” (P13)  “*App, if the step is not complicated, then it will be easy, just press a button and read all the information*.” (P12)  “*If this webpage has the same function about knee pain… then sometimes people do not need to come… Less medical fees, save the government some money… reduces your workload*.” (P22)  **Physical interaction is preferred and irreplaceable** (n = 8)  “*Technology, no matter what, cannot be compared. When we communicate with others, we have our tone, mannerisms, even charisma: you can feel it. It (technology) cannot be compared to physical interactions and its effects, definitely not comparable*.” (P13)  “*Bobian then I will use the app. But if can, I prefer face to face. I think the human touch is missing in the apps, you have no human interaction on whether I'm doing right or wrong*.” (P20)  “*Convenient though this is, I would prefer face-to-face, personal contact. Talking to them over video call is not the same as being there with them... I think personal interaction - I see you face to face, I talk to you - is better, more personal. The screen is a bit cold and detached sort of feeling*.” (P14) |
| Domain: **Implementation** **(Context)**  Perspective: **Healthcare professionals**  Theme 1: **Quick-fix and passive attitude towards treatment due to socioeconomic reasons**  Theme 2: **Pre-existing beliefs, mindsets, expectations and misinformation** | **Patients expect quick-fixes with high expectations** (n = 9)  “*Those that just generally want a quick fix solution and medication or surgery then the inter-disciplinary component proposed to them hasn’t been very well accepted… it’s almost like having a cold that makes you feel unwell, you want to get rid of it or try and solve it. So similarly with pain I think it would be a similar concept for the patient*.” (Psy01)  “*Another reason might be… I just want a quick fix, ‘I just want the doctor to give me an injection’ then I don’t need effort. But the whole thing is actually a lack of knowledge*.” (PT02)  “*I can tell you they wish for something that is pain-free, non-invasive, low risk, and will solve their problems. But for this condition, there is no quick fix, no magic bullet… I think a lot of patients expect things should be done for them, that healthcare should take care of them*.” (Doc03)  “high expectations: wanting to go back (to normalcy), to be as strong and fit as before, and it may be due to their role in the family or society. Maybe they used to be high fliers, used to be very active, play sports…and that loss of function then matches the loss of status… Generally, as humans, when we seek treatment, we would always subconsciously expect 100%... I had patients that have said “I’ve achieved all my goals, I’m 99.99% better than before, I’m so grateful! But can you tell me how to improve further? I need to be back to more than a 100% before”… in terms of treatment expectations, they expect a certain level of success, maybe within a week or so, being able to go back to normal, not having much pain, etc. And when those are not met then it ends up looking like treatment failure. These patients are the ones that from the start we’ve already told them it’s unrealistic to go back exactly to 100%... *I think many of them, once they get better, forget how bad it was and seek further improvement, which is probably, at the moment, not the most realistic, in terms of what we have available.*” (PSY01)  **Patients’ passive attitude and expectation towards treatment** (n = 7)  “*They feel they need the therapist to do something for them to get better; they have developed a very passive approach towards management of their condition. This faulty belief system may cripple them from becoming fully independent*.” (PT04)  “*It will be good to change their mindset to be more active. We can work together as patient and physio, but the patient must actively engage with us... sometimes they can be quite passive and just want to come here for a heat pack, but they are not actively engaged in the community*.” (PT05)  “*One is mindset that ‘healthcare professionals should take care of everything; I don’t need to bother’... For example, physio wise, there is that patient's belief that ‘I need to go to a facility or location... I must follow a trained professional before I can do it by myself’. We’re not expressing that kind of platform for them to be more independent*. (Doc03)  **Logistical issues: Availability of self and caregiver and treatment cost** (n = 8)  “*Time of work if they are still working and getting family involved. Whether they want to get family involved, and the ability to retain them. If they have difficulty for transport like they can’t go by themselves or they require family members to go with them, that’s pivotal*.” (Doc03)  “*If the elderly need someone to bring them, then it’s difficult for them because their children may be working. Those are the two major reasons: costs and nobody to bring them… if they are still working, they may not have time to come... Providing them channels to get access to physiotherapy is easy, but everything about the system is that you have to pay for it, and cost is always a big issue*.” (Doc05)  *“… the kind of support they have to continue working, endure the pain or if they can take time off and depend on family support. I think there’s a lot of factors involved and we can’t really pinpoint as it’s quite individualized*.” (PT03)  **Beliefs and misinformation** (n = 7)  “*They think they know what they should be eating… they must have heard from their family and friends what was the right thing to eat or the internet or something like that*.” (Doc01)  “*Some feel that ‘my leg is already painful yet you still ask me to exercise, what if it makes the pain worse?’. They don’t understand their condition that actually the appropriate exercise would be prescribed*.” (PT02)  “*Many holds the myth that "I walk all day, that’s exercise to me"… A lot of them they have misconception about exercise like, "I go buy grocery, walk around, do my chores, mopped the floor, are those are not exercises?" A lot of them do not understand the concept of exercise.*” (MSW01)  **A need to educate to change mindsets and expectations** (n = 6)  “*Talking to them and understanding them in the community, you can provide prevention, which is always better than cure... At an early stage, by changing their behaviour when there is less pain, maybe they will be more compliant by changing their mindset*.” (PT05)  “*At least they have an understanding of their condition, expectation of how physiotherapy can help their condition, so we can tailor their expectation and guide them towards more active management*.” (PT04)  “*I wouldn’t say a huge percentage of people that expect a miracle. Most of the time we will already educate them on… how fast they can recover. The first session is important in setting their expectations right*.” (PT03) |
| Domain: **Implementation** **(Context)**  Perspective: **Patients**  Theme 1: **Resignation to OA**  Theme 2: **Mixed perception to acupuncture** | **OA is passively naturalised and accepted as a result of aging** (n = 21)  “*What I have heard of, I have friends who have this kind of knee problems, so I accepted as part of ageing, wear and tear*.” (P14)  “*Normally* *they* (colleagues and family) *say this is natural ageing process, so it seems like unavoidable when we reach certain age this knee problem will occur somehow. So just take it at face value…I think maybe the joint already wear out... I believe probably is due to wear and tear, so I have to live with this problem*.” (P12)  “*I just live with it. at present I just accept… cause not much things can be changed*” (P04)  “*When they (doctors) say is early degeneration, we think that well it’s like this, so you just have to live with it*” (P05)  **Mixed perception and evaluation of acupuncture** (n = 13)  “*It never crossed my mind… It didn’t actually help, TCM I don’t think it will help.*” (P05)  “*One thing I hate needles so ask me to go TCM. Aiyo scary lah! Don’t want, don’t want!*” (P13)  “*I wouldn’t have gone because* *I just don’t believe in TCM… I don’t know if they* (TCM physicians) *gone through any course like a doctor who has gone through medical school… TCM to me, it's just like taking herbs and this and that, not proven, so I don’t really go.*” (P14)  “*I actually I went to… the Chinese* sinseh (physician) *to do all those acupuncture, for about 7-8 times. But it still doesn't seem to improve.*” (P20) |
| Domain: **Mechanism of impact**  Perspective: **Patients**  Theme 1: **Active acceptance, self-efficacy, and self-management capability**  Theme 2: **Learning, motivating, empathizing, and socializing in a group**  Theme 3: **Intervention synergy** | **Active acceptance, self-efficacy, and self-management capability** (n = 11)  “*The main purpose of the psychology classes is to help us not be too dispirited, if you suddenly face a problem, you can use another angle to look at the problem. You won't expect to go back to the original state where your knee is fine because the cartilage is gone and cannot be restored. You need to face it, acknowledge the reality so that you won't feel so dispirited.*” (P06)  “*It's all on your own self-discipline... Every morning I wake up must really do exercise. Last time, faster la, no time no need to do. Now quite consistent… if nobody tell you, you also don’t bother, don’t do consistently, you might skip, it will not help. Consistent exercise will help really, I can feel that*.” (P01)  “I recalled the psychologist said, "if you fall, how do you pick yourself up, and not let yourself hit rock bottom”. During the class, Dr XXX taught us what we should do, to rest, to hold, etc. Hence, you know you can use these technique… I employed those techniques. Although now I am not as good as I was before, I feel I can recover back to that level.” (P03)  **Benefits of group intervention: learning, motivation, empathy, and socialization** (n = 18)  “*There were other people there who were shared their experiences. It would make you feel like there are others like that as well, some could even be worse. I felt some sort of encouragement. You would feel like "you are not alone; you are not the only one", there are others who go through this treatment together with you.*” (P03)  “*Quite interesting to know, understand each other’s conditions, and to encourage each other… Motivating! You have friends to talk to, you can interact, you can understand each other in terms of the pain*.” (P05)  “*Group motivation is better. Because you compare to each other… Peer pressure. Group motivation is good, even for exercise. Group motivation is good because we do in a group, whereas sometimes at home, you can procrastinate*.” (P15)  “*We will help to motivate each other because we are all patients. We are not healthy people, so mutual support helps*.” (P18)  **Synergy among EDU-PSY-NND** (n = 7)  “*It* (PSY) *gave me a lot of encouragement so that I didn’t feel so hopeless. I learned not be so depressed… I learned what is good and what not to eat. They taught me how to lose weight… I don't know how much weight I have lost but my clothes now are not as tight as last time*”. (P17)  “*About how to manage and not to expect the leg to be same condition as previous, accept the present... do things in small steps… You don't only just learn, you have to put into practice... Dietician shared with us the things to look out for… the food we should eat… As long as I can walk, I don't limp any more, the pain is there I know how to manage. And like Dr XXX says, the flare may occur, if it happens, how I manage it? That's it, I accept, I think acceptance is important. I know how to set my own expectation that I cannot be like last time*.” (P20)  “*No matter my situation or state of mind, I must not dwell on it. I need to really walk out of it, don’t coop myself up at home. It helped to change my way of thinking…* (NND) *has helped me to make many dietary changes. In the past, I would have a few biscuits and a cup of Milo for lunch. Later, when I learned that it is wrong and a lack of protein is bad for health…* (now) *I would have proper meals but I will still snack*.” (P21) |
| Domain: **Maintenance**  Perspective: **Patients**  Theme 1: **Improvements motivated exercise and physical activity consistency, habit, and goals**  Theme 2: **Perceived lack of time, inertia, and complacency weakens self-maintenance** | **Experienced improvement in pain and function** (n = 16)  “*I don’t feel the pain when I walk... I still do exercise. Of course, I feel better or else I will feel pain. Sometimes I can feel the pain, it's on and off, not always pain... at least 80% back* (to normal)*… it doesn’t really disturb me that much, so I tend to forget.*” (P01)  “*At first improve, then slowly, "eh?! How come no pain already?!” Even when I walk people will notice* (there is an improvement). *Last time when I walk my, friends and colleagues say, "aiya your leg still not so good"… My knee is okay, very flexible, I just hope that I can maintain like this… after joining the programme, I can kneel down on the pew… I feel good!*” (P02)  “*I am not guaranteed recovery after completing the program. Gradually I felt I was actually doing very well. In the middle* (of the intervention period)*… I was at my best. I felt that at that time I was fully recovered. That was my tiptop period… It has been a long time since I felt that great.*” (P03)  **Exercise and physical activity: self-maintenance, consistency, and progression** (n = 14)  “*I joined park exercise… 香功* (xiang gong). *Sometimes alternate days, sometimes every day... I do my physiotherapy, I bring my* (physiotherapy) *paper there, I do my own physiotherapy. After that* xiang gong, *I feel that it's very good, my breathing is very good... I join yoga every Thursday, 2 to 4 o clock.*” (P11)  “*It helps you to strengthen the muscle, manage the knee pain, so you got muscle, all the exercises are good…*  *I can do more walking, can do more activities. I become less inactive*… *I have been cycling for one year already. So far, I clocked 800km. Because cycling has no impact on my knees. I can push, if upslope's more difficult... I just want do something to exercise. If I want to walk, I can't. Jogging, no way, because my knee can't take it… I believe it's* (cycling) *good... no pain then I want to do more*.” (P19)  “*I also now started to do more walking… about 45 minutes to one hour. That will cover about 4km. I can do all these now… I don’t golf anymore so I do the rest of the other exercise to tone my body up. Every day I walk, cycle, and do all the stretching that I learn at St Luke’s. St Luke’s also teach us a few exercise… stretching exercises to strengthen the thigh muscle, hamstring and calf.*” (P07)  **Barriers to self-maintenance: perceived lack of time, inertia, and complacency** (n = 11)  “*I still have to do housework at home… I even hardly watch television. I usually have other things to do… Not much time, I am quite busy*.” (P22)  “*On my own is very lazy, say I will do but never do… I tell myself there is no pain*.” (P20)  “*For the first few months, yes. But after that**, I almost forget about it because there's no pain, seems like there's no motivation to do the exercises*.” (P12)  “*After I was discharged,* *I sort of gradually fell back and didn’t bother with the exercises because the pain was not bothering me so much, I could put up with it*.” (P14) |
